# Role of Phenylpropanoids and Flavonoids in Plant Resistance to Pests and Diseases

**DOI:** 10.3390/molecules27238371

**Published:** 2022-11-30

**Authors:** Marie-Louisa Ramaroson, Claude Koutouan, Jean-Jacques Helesbeux, Valérie Le Clerc, Latifa Hamama, Emmanuel Geoffriau, Mathilde Briard

**Affiliations:** 1 Univ Angers, Institut Agro, INRAE, IRHS, SFR 4207 QUASAV, F-49000 Angers, France; 2 Univ Angers, SONAS, SFR 4207 QUASAV, F-49000 Angers, France

**Keywords:** specialized metabolites, plant defense, resistance mechanisms, biotic stress

## Abstract

Phenylpropanoids and flavonoids are specialized metabolites frequently reported as involved in plant defense to biotic or abiotic stresses. Their biosynthetic accumulation may be constitutive and/or induced in response to external stimuli. They may participate in plant signaling driving plant defense responses, act as a physical or chemical barrier to prevent invasion, or as a direct toxic weapon against microbial or insect targets. Their protective action is described as the combinatory effect of their localization during the host’s interaction with aggressors, their sustained availability, and the predominance of specific compounds or synergy with others. Their biosynthesis and regulation are partly deciphered; however, a lot of gaps in knowledge remain to be filled. Their mode of action on microorganisms and insects probably arises from an interference with important cellular machineries and structures, yet this is not fully understood for all type of pests and pathogens. We present here an overview of advances in the state of the art for both phenylpropanoids and flavonoids with the objective of paving the way for plant breeders looking for natural sources of resistance to improve plant varieties. Examples are provided for all types of microorganisms and insects that are targeted in crop protection. For this purpose, fields of phytopathology, phytochemistry, and human health were explored.

## 1. Introduction

Plant secondary metabolites are involved in various biological functions and play a role in plant interactions with their environment, particularly under biotic and abiotic stresses. While some of these metabolites play a fundamental role in the attraction of pollinators and in chemical ecology, others are involved in coping with stressful stimuli, as reviewed in [1,2,3,4,5,6]. They were not considered essential for plant growth and development when they were first discovered and were qualified as “secondary”. Nevertheless, nuances in the definition of secondary metabolites have emerged over the past two decades. Because of their pivotal role in the plasticity and response of plants to various environmental stimuli, some authors rather refer to them as “specialized” metabolites, while “central” is used for primary metabolites [7,8,9]. Moreover, the advent of high-throughput sequencing has allowed the publication of the genomes of several species and highlighted that the genes involved in the biosynthesis pathways of these metabolites occupy a significant place in a large array of genomes [10]. As we fully adhere to the concept of high importance of these metabolites, the term “specialized metabolites” will be used throughout this paper.

Metabolomics and functional genomics technologies have accelerated the large-scale exploration of plant specialized metabolites and the key enzymes involved in their biosynthesis [11,12]. The objective of the present review was to compile data about phenylpropanoids and flavonoids because of their wide range of biological activities and particularly their significant involvement in numerous mechanisms of plant adaptation to the environment.

The phenylpropanoid pathway (PPP) results in the accumulation of many families of compounds, such as phenylpropanoids, flavonoids, lignins, monolignols, phenolic acids, stilbenes and coumarins [13]. The flavonoid family includes subfamilies of molecules classified according to their structure, e.g., flavones, isoflavones, anthocyanidins, flavonols, flavanols, flavanones, aurones or chalcones. Each subfamily comprises a large diversity of molecules as a result of various conjugation processes through *C*- or *O*-methylation, sulfation, or glycosylation [14,15,16].

In stress-free conditions, flavonoids play a role in the development of plant reproductive organs and seeds, such as pollen tube germination and growth or seed maturation, dormancy and longevity [17,18]. They are also involved in plant attractiveness to pollinators through the color or scent they confer to flowers [19]. Finally, they also play a role in plant–microorganism communication for the establishment of symbiosis, such as in legume–rhizobium interactions during nodulation [18,19].

**Figure 1:**
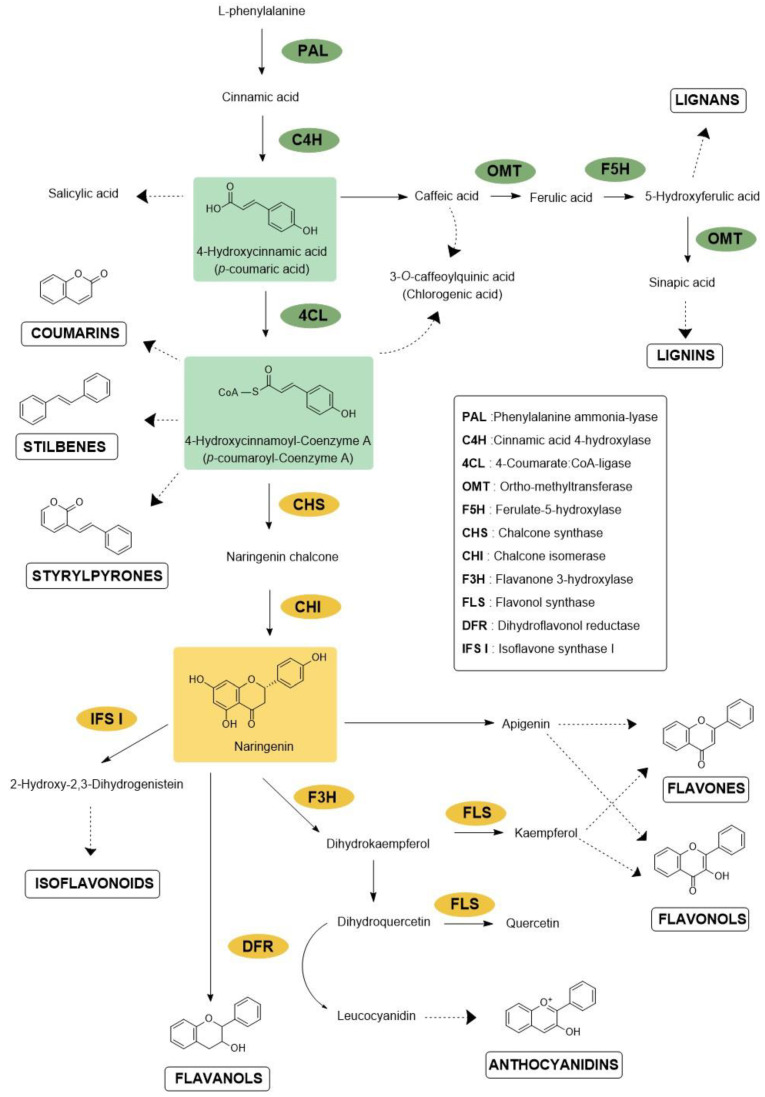
Main steps of the phenylpropanoid and flavonoid pathways. Enzymes mentioned in this paper are shown in green and yellow for central phenylpropanoid pathways and flavonoid biosynthesis, respectively. Complete arrows refer to one step in the biosynthetic pathway, whereas dashed arrows represent undetailed pathways leading to one molecule or molecule subfamilies.

In adverse abiotic conditions, they can mediate defense responses. For instance, under water stress, plants have to deal with concomitant oxidative stress caused by reactive oxygen species (ROS) to prevent cellular damage. For this purpose, high antioxidant activity could be necessary to limit lipid peroxidation of cell membranes [20,21]. This may be obtained through the upregulation of genes involved in the phenolic flavonoid biosynthesis described in Figure 1. For example, in *Chrysanthemum morifolium* L. cultivars exposed to water stress, genes encoding enzymes phenylalanine ammonia-lyase (*PAL*), chalcone isomerase (*CHI*) and flavanone 3-hydroxylase (*F3H*) were upregulated, leading to an increased production of antioxidant flavonoids [22]. Biosynthesis of caffeic acid derivatives and flavonoid glycosides was also strongly enhanced during salt and UV stresses [23]. The mutation of several genes coding for a *Myeloblastosis (MYB)* transcription factor, a chalcone synthase (*CHS*) and a few chalcone isomerases was reported to alter the freezing tolerance of *Arabidopsis thaliana* (L.) Heynh. [24]. Under excess of solar radiation, flavonoids strongly accumulate in leaves and glandular trichomes of *Phillyrea latifolia* L. [25] leading authors to suggest their protective role in the integrated mechanisms of acclimation of *P. latifolia* to excessive light.

Finally, in a context of biotic stresses, plant defense may be mediated by the action of flavonoids and phenylpropanoid compounds acting indirectly as signaling molecules, or directly through the toxic effect of phytoanticipins (constitutively accumulated active compounds in plant tissues) and phytoalexins (newly synthesized active compounds following pathogen detection) [26,27,28]. Their accumulation or the importance of the expression of genes involved in their biosynthesis has indeed been demonstrated in regards of resistance to biotic stresses [29,30,31,32]. This has been described in the literature from two points of view. On the one hand, research focusing on basal defense addressed the different types of constitutive defense mechanisms, from physical to chemical barriers. Cell wall reinforcement involving phenylpropanoid derivatives is one of them [30,32,33,34,35]. As an example, the abundance of phenylpropanoids in maize (*Zea mays* L.) grain pericarps was thought to limit disease symptoms in genotypes resistant to *Fusarium graminearum* and *Fusarium verticillioides,* as well as against maize weevil (*Sitophilus zeamais* (Motsch.) [35,36,37]. On the other hand, research focusing on induced resistance investigated the potential toxicity of these metabolites that may suppress or limit the pathogenicity of invaders. Although the study of this direct effect played by bioactive compounds against pathogens has been promoted by a rising interest in deciphering the molecular dialogue between the host and the pathogen, these mechanisms are still poorly described in plant science. In contrast, the biological activity of plant-derived compounds—especially flavonoids—on human pathogenic microorganisms has been notably investigated in the field of drug development [38,39]. Moreover, potential intracellular targets of some flavones have been discovered when searching for natural anti-inflammatory compounds [40].

In order to help breeding for plants resistant to pests and diseases or plants receptive to biopesticides, this review covers the state of the art on the molecular and mechanistic diversity of phenylpropanoid or flavonoid derivatives potentially involved in plant resistance to biotic stresses. This is presented according to the nature of the targeted pathogen, including the highlights of the findings in human health research.

## 2. Protection against Microbes

### 2.1. Bacterial Targets

A wide range of studies have explored the involvement of the PPP in mediating a response to phytopathogenic bacteria. For example in the tobacco (*Nicotiana tabacum* L.)–*Pseudomonas syringae* pathosystem, an infection-induced increase was shown for the flavonoids and other phenylpropanoid derivative content, especially regarding coumaric acid content [32]. Similarly, flavonoid glycosides and hydroxycinnamic acid production significantly increased in orange leaves (*Citrus sinensis* L.) infected by *Candidatus liberibacter asiaticus* [29]. In potato (*Solanum tuberosum* L.) tubers, rutin (quercetin-3-*O*-rutinoside) (Table 1) and nicotiflorin (kaempferol-3-*O*-rutinoside) were shown to be related with resistance to *Pectobacterium atrosepticum*, a necrotrophic bacterial pathogen [41]. Finally, resistance to *Erwinia carotovora* subsp. *carotovora* of transgenic potato tubers accumulating a high content of pelargonidin-3-*O*-rutinoside-5-*O*-glucopyranoside and peonidin-3-*O*-rutinoside-5-*O*-glucopyranoside both acylated with *p*-coumaric acid, was double that of untransformed plants with lower amounts of these anthocyanins (Table 1) [42].

In a context of plant protection, in order to develop biological alternatives to synthetic phytoprotectants, the biological actions of phenylpropanoids have been largely investigated through in vitro tests. However, according to [43], an efficient protection may probably not arise from any significant toxicity towards the microorganism, but rather from defense elicitation of the host. Therefore, to reach an efficient pest management, we suggest exploring both the direct or indirect modes of action of these compounds. As a consequence, there is a need to delve into the question of their biosynthesis and targets.

By studying the postinoculation transcriptomic shift, some genes encoding enzymes at a crucial step in the PPP were found to rapidly respond, whereas others were induced much later in the onset of the response to infection [31,32]. In soybean (*Glycine max* (L.) Merr.) infected with *Pseudomonas syringae*, *CHS*, *F3H* and isoflavone synthase I (*IFS I*) genes were among the earliest to be activated [31]. In tobacco infected with the same bacterium, *PAL*, cinnamic acid 4-hydroxylase (*C4H*), 4-coumarate:CoA-ligase (*4CL*), some ortho-methyltransferases (*OMT*) and ferulate-5-hydroxylase (*F5H*) genes were the most expressed [32]. At this stage, although the discovery of these potential genes of interest may pave the way for the development of new plant varieties resistant to bacteria, little is known about the biological targets of the enzymes related to these genes. Plant-oriented literature only reports that some PPP compounds can target vital functionalities of cellular processes and jeopardize bacterial survival without details about the mechanisms implemented. For example, fragarin isolated from strawberry (*Fragaria ananassa* (Weston) Duchesne ex Rozier) leaves caused cell death by disrupting cell membrane integrity in *Clavibacter michiganensis* [44,45]. Interesting data from research in medical microbiology may nonetheless provide leads, as a number of authors have proposed an elaborated description of the mechanism of action for phenylpropanoids to find natural antibiotics for human health [46,47]. Thus, the antimicrobial action of flavonoid glycosides isolated from the aerial parts of *Graptophyllum grandulosum* Turrill—chrysoeriol-7-*O-β*-d-xylopyranoside, luteolin-7-*O-β*-d-apiofuranosyl-(1→2)-*β*-d-xylopyranoside, chrysoeriol-7-*O-β*-d-apiofuranosyl-(1→2)-*β*-d-xylopyranoside, chrysoeriol-7-*O-α*-l-rhamnopyranosyl-(1→6)-*β*-d-(4″-hydrogenosulfate) glucopyranoside and isorhamnetin-3-*O*-rutinoside (Table 1)—was reported to cause cell lysis in *S. aureus* due to alteration of membrane permeability, with a minimum inhibitory concentration (MIC) ranging from 4 to 8 µg.mL^−1^ [48]. This membrane fluidity alteration may arise from the upregulation of genes responsible for a rearrangement of the membrane fatty acid proportions, as observed in *S. aureus* and *E. coli* exposed to a relatively low concentration of naringenin (Table 1) (with a MIC of 1.84 mM and 3.64 mM respectively) [49]. The physicochemical properties of this class of compounds are involved in their ability to cross the bacterial wall and lipidic membranes to reach their intracellular targets [50]. A low capacity of cell penetration could explain why the cytotoxicity of some molecules may go unnoticed in the framework of in vitro tests on a whole-cell scale, in spite of an apparent effect observed on isolated cellular components [51].

The cell walls of Gram-positive and Gram-negative bacteria are structurally divergent. The wall of Gram-positive bacteria comprises a cytoplasmic membrane underneath a periplasmic space mainly composed of peptidoglycans, whereas Gram-negative bacteria have an additional membrane on the outer side, coated by lipopolysaccharides, rendering bacteria of this type rather less permeable to relatively more hydrophobic compounds. Therefore, this could explain the discrepancy in the antibacterial activity of the same active compound. This was observed with naringenin [49] whose growth inhibition was more perceptible on a Gram-positive (*S. aureus*) than a Gram-negative bacterium (*E. coli*). Chlorogenic acid (Table 1) can cause dismantling of the outer membrane in *Shigella dysenteriae* (Gram-negative). It was hypothesized that its acidic carboxyl group may chelate stabilizing cations in the lipopolysaccharide layer, which further disrupts the outer membrane structure [52]. Although it is clear that a certain level of lipophilicity of the compound is required to interact with and pass through the cell membrane [53,54], manipulating other properties—e.g., by adding sugar moieties—can improve the interaction with enzyme active sites, compared to aglycones [15,40]. Therefore, studying the structure–activity relationships of the specialized metabolites in regard to the physical barrier of the bacteria is undoubtedly essential in searching of efficient antibacterial compounds.

Compounds with better penetrability exhibit stronger effects on key functionalities in the intracellular milieu. First of all, the electron transport respiratory chain, and thus cell survival, can be dramatically affected. This has been demonstrated in a case study on *Micrococcus luteus*: for licochalcones A and C (Table 1), two retrochalcones isolated from Chinese licorice (*Glycyrrhiza inflata* L.) roots compromised the enzymatic activity of the NADH-cytochrome C reductase [51]. In addition, the antibacterial activity of the compound of interest may consist in preventing cell proliferation, through a direct interference with cell division processes. In *E. coli*, the polymerization of an important cytoskeletal protein FtsZ—required for cytokinesis—was hindered by the action of chlorogenic acid, leading to inhibition of cell division (Table 1) [55]. A molecular modeling study suggested that chlorogenic acid made hydrogen bonds and hydrophobic interactions to various residues of this protein, hence altering its conformation and disabling the GTPase activity preceding polymerization [55].

These few discoveries illustrate the action of molecules from the PPP family at different subcellular levels, targeting essential functionalities and fundamental structural components of the bacterial cell. The investigation could be transposed to plant-pathogenic bacteria to support previous hypotheses on plant disease resistance through the biological activity of phenylpropanoid derivatives.

### 2.2. Fungal Targets

Since the early 2010s, the accessibility of metabolomic tools has hugely favored the identification of specialized PPP metabolites correlated with plant resistance to fungi and oomycetes [56,57,58,59,60]. *O*-glycosylated flavonoids are the most frequently reported in such host–pathogen interactions. The mechanistic studies published so far followed an overall trend emphasizing a quantitative aspect and/or a spatiotemporal dimension of phenolic derivative accumulation in plant tissues associated with resistance to fungi.

Quantitative aspects can be illustrated by a case study on carrot (*Daucus carota* L.) leaves. Genotypes more resistant to *Alternaria dauci* contained significantly higher levels of feruloylquinic acid, as well as 4′-*O*- and 7-*O*-glycosides of apigenin, luteolin (Table 1) and chrysoeriol, compared to susceptible genotypes [57]. Such differences in PPP metabolites between resistant and susceptible genotypes may be found in numerous other pathosystems providing support for plant breeders. Nevertheless, with the same compounds, an opposite correlation can be found between metabolite contents and disease resistance depending on the pathogen. This was specifically observed in potato tubers enriched in rutin and nicotiflorin that turned out to be resistant to *Pectobacterium atrosepticum*, as mentioned above, but at the same time susceptible to *Phytophthora infestans*, a biotrophic fungal pathogen [41]. A systemic approach is therefore required to make sure that breeding for an increased content of a specific specialized metabolite involved in resistance to one pathogen will not decrease resistance to another one.

Spatial aspects can be exemplified by maize—*Fusarium graminearum* and maize—*Fusarium verticillioides* pathosystems, where a high content in ferulic acid in the grain pericarp was linked to a lesser disease extent in resistant genotypes [36,37]. Such a specific localization of the defense metabolite seems to be a very strategic way to prevent pathogen invasion. For example, in cotton (*Gossypium hirsutum* L. and *Gossypium barbadense* L.), catechin and gallocatechin (Table 1) were predominant near the *Verticillium dahliae* infection site in the vessels, creating a toxic environment that confined the pathogen to the vessel lumens [61]. This local accumulation contributed to prevent the systemic spread of the vascular disease through the formation of tyloses. This mechanism was also observed in grapevine (*Vitis vinifera* L.) defense against *Phaeomoniella chlamydospora* and *Phaeoacremonium* species [62]. Similarly, the resistance of barley (*Hordeum vulgare* L.) to mildew (*Blumeria graminis*) was attributed to the accumulation of light-absorbing compounds—suggested to be phenylpropanoids—in the papilla of the coleoptiles [63].

Temporality can be illustrated by the resistance of the date palm tree (*Phoenix dactylifera* L.) to fungal diseases caused by *Fusarium oxysporum*, likely driven by a quantitative differential in 5-*O*-caffeoyl-shikimic acid content, particularly at physiological stage 3 (ripening of dates) [64]. The phenotypic contrast between resistant and susceptible cultivars to a fungal disease is often attributed to an early or constitutive availability of the compound of interest in the tissues leading to an efficient defense response, e.g., preformed chlorogenic acid in tobacco plants resistant to *Cercospora nicotianae* [65]. Similarly, studies on the barley (*Gibberella zeae*) pathosystem led to the identification of 194 metabolites constitutively present in a resistant genotype and significantly more accumulated than in a susceptible one [56]. Among them were kaempferol-3-*O*-rhamnopyranoside, naringenin-7-*O*-glucopyranoside, kaempferol-3-*O*-rhamnopyranoside-7-*O*-glucopyranoside, kaempferol-3-*O*-glucopyranoside-7-*O*-rhamnopyranoside, and kaempferol-3-*O*-sophoroside-7-*O*-rhamnopyranoside. Such a constitutive accumulation of flavonoids in the context of disease resistance in various plant–fungus pathosystems is now becoming a general trend described in many studies [56,66,67,68]. In addition to an early or constitutive synthesis of the defense compounds, their maintenance at a high concentration over time, i.e., as long as the disease pressure is high, is equally important [67]. Therefore, plant resistance to fungal diseases is not only more complex than a simple quantitative differential between resistant and susceptible genotypes at the time of infection, but its durability also depends on the stability of metabolite contents in the tissues over time. Thus, according to the development cycles of the disease and those of the plant, an efficient metabolic ratio must be maintained.

A multidisciplinary approach combining reverse genetic tools with biochemical characterization of the resulting proteins has led to a better understanding of the biosynthesis of phenylpropanoid derivatives mediating fungal disease resistance. Firstly, due to the upstream position of the *PAL* gene in the PPP, modifications would deprive the plant from the biosynthesis of a lot of compounds driven by downstream genes in the pathway. A tobacco *PAL* mutant (*PAL*-suppressed YE-6-16 transformant) exhibited a rapid expansion of lesions after infection by *Cercospora nicotianae,* whereas *PAL* gene overexpression resulted in reduced disease symptoms [65,69]. In Arabidopsis thaliana, inactivation of a gene encoding a *CHS* led to a decreased anthocyanin content and lower resistance to *Verticillium dahliae* [70]. In contrast, overexpression of *CHS*-, *CHI*- and dihydroflavonol reductase (*DFR*-) encoding genes in flax (*Linum usitatissimum* L.) was correlated with increased resistance to *Fusarium* species through an increased flavonoid content [71]. Overexpression and mutation of an *R2R3 MYB* transcription factor clearly impacted resistance to *Dothiorella gregaria* in poplar (*Populus tomentosa* Carr.) through enhanced and decreased proanthocyanidin content, respectively [72]. As supplementary evidence, chemical inhibition of a *CHS* enzyme and downregulation of the corresponding *CHS* gene in cucumber (*Cucumis sativus* L.) resulted in nearly complete suppression of induced resistance towards *Podosphaera xanthii* [73].

The antifungal activity exerted by phenylpropanoids, and flavonoids has been investigated, corroborating the metabolomic and functional genomic-driven hypothesis on their link to disease resistance. Flavonoids extracted from the needles of *Picea neoveitchii* Mast., used at 1 mg·mL^−1^, exhibited very interesting antifungal activities: kaempferol-7-*O*-(2″-*E-p*-coumaroyl)-*α*-l-arabinofuranoside exhibited strong activity against *Fusarium oxysporum* with a relative inhibitory percentage of 108.1%, while 5,7,4′-trihydroxy-3,8,-dimethoxy-6-*C*-methylflavone, 5,8,4′-trihydroxy-3,7-dimethoxy-6-*C*-methylflavone, 7-methoxy-6-*C*-methylkaempferol and kaempferol-7-*O*-(2″-*E-p*-coumaroyl)-*α*-l-arabinofuranoside were active against *Rhizoctonia solani*, with 49.5%, 53.3%, 95.3% and 49.5% relative inhibitory percentages, respectively, (Table 1) [58]. These compounds were as active as carbendazim, a synthetic chemical fungicide used against these two pathogens. Other flavonoids, such as eriodictyol, homoeriodictyol, dihydroquercetin, and luteolin (Table 1) isolated from *Ficus sarmentosa*, var. henryi (King) Corner, were effective against pathogenic fungi, e.g., *Fusarium graminearum* and *Septoria zeicola*. Among these flavonoids, luteolin showed the strongest inhibitory activity, with half-maximal inhibitory concentration (IC_50_) values of 56.38 and 81.48 mg·L^−1^ to each fungus, respectively [74]. Finally, in an in vivo assay, cherry tomatoes sprayed with laurel (*Laurus nobilis* L.) oil containing about 44% of eugenol and 30% of cinnamaldehyde (Table 1) were less infected by *Alternaria alternata* after 5 days of storage at 25 °C than control without oil. More precisely, the proportion of decayed tomatoes treated with 1 mg·mL^−1^ of laurel oil was reduced by 86.4% [75].

Despite all these investigations on biosynthesis and targets, little is known about the mode of action involved in the described fungicidal activities. Some studies have assessed the effect of phenylpropanoid derivatives on the integrity of plant fungal pathogens. Among them is the study of essential oil from laurel leaves mentioned above: fungicidal activity was evidenced with invaginations and folds in the cell wall of the fungus and drastically reduced sporulation.

Medical research suggested another mechanism: a high concentration of a bioactive caffeic acid derivative like chlorogenic acid in the extracellular environment was efficient to disrupt the lipid membrane of *Candida albicans*, *Trichosporon beigelii* and *Malassezia furfur*, leading to ion leakage and break of the intracellular equilibrium (Table 1) [76]. Apigenin (Table 1) isolated from the leaves of *Aster yomena* (Kitam.) Honda had the same effect on *C. albicans*: it caused intracellular calcium and potassium leakage and led to osmotic imbalance [77]. The capacity of some compounds to cross cell boundaries suggests that they might reach and interfere with nuclear components. Despite the lack of clear evidence of a nucleic acid–flavonoid interaction, apoptosis-associated DNA fragmentation and chromatin condensation was observed in *Candida glabrata* following treatment with 4 µg·mL^−1^ of glabridin (Table 1), an isoflavan mainly found in *Glycyrrhiza glabra* L. roots [78]. These mechanisms may also occur in plant fungal pathogens and should be explored by plant prebreeders.

### 2.3. Viral Targets

Plant exposure to viral agents activated salicylic acid (SA) biosynthesis and induced the biosynthesis of other defense metabolites from the PPP to initiate systemic acquired resistance (SAR). This was shown for the sugarcane mosaic virus that causes dwarf mosaic disease on maize [79]. As it is hosted by a broad range of economically important plant species, such as tobacco and tomato (*Solanum lycopersicum* L.), specific attention has been paid to the tobacco mosaic virus (TMV) in the exploration of the antiviral activity of candidate specialized metabolites [80]. A more precise study pointed out that while 5-*O*-caffeoylquinic acid and quercetin abounded at the TMV infection site in tobacco leaves, kaempferol was predominant in a more remote part of the plant that exhibited SAR (Table 1) [11].

Most of the publications pointed out the role of quercetin and kaempferol (Table 1) in triggering the defense response of the host plant, rather than a direct action on viral particles, e.g., in the *Datura stramonium* L.-TMV and *Chenopodium amaranticolor* H.J. Coste and A.Reyn.—TMV pathosystems [81]. Other studies mention a correlation between the metabolite content and plant resistance to viruses, without further determining whether they are potentially harmful or not to viral particles.

In medical research, one of the earliest works carried on murine leukemia viruses (MLVs) and human immunodeficiency viruses (HIVs) revealed that 1 µg·mL^−1^ and 2 µg·mL^−1^ of 5,6,7-trihydroxyflavone (baicalein) inhibited the activity of their respective reverse transcriptases by 90% (Table 1) [82]. A similar trial on HIV showed the same trend, with approximately 90% of reverse transcriptase inhibition at 200 µg·mL^−1^ of hinokiflavone and robustaflavone (Table 1) isolated from *Rhus succedanea* L. [83]. This surely brings evidence that phenylpropanoid derivatives could limit viral reproduction within the host, although the way they inhibit the biological function of this strategic enzyme remains unclear. These observations need to be transposed to the framework of plant pathology studies to determine the putative direct effects of these compounds on plant viruses.

## 3. Protection against Insects

Among macroscopic pests of cultivated plants, insects are a concern, not only because of their direct damaging effect mainly linked to the herbivorous activity of their larvae but also because of indirect damage through their ability to transmit microbial pathogens to the host plant.

### 3.1. Herbivore Targets

Efforts have been made to explore the natural defense mechanisms of plants against herbivores. The defensive compounds are either produced constitutively or in response to plant damage, and affect feeding, growth, and survival of herbivores. In addition, plants also release volatile organic compounds that attract the natural enemies of the herbivores, as reviewed in [84]. Phenylpropanoids and flavonoids are mentioned among these defensive compounds preventing plants from insect invasion. For example, chlorogenic acid and feruloylquinic acid (Table 1) discriminate resistant and susceptible genotypes of chrysanthemum (*Dendranthema grandiflora* (Ramat.) Kitam.) to thrips (*Frankliniella occidentalis*) with higher amounts of both molecules in thrip-resistant genotypes [85]. Similarly high contents of quercetin, chlorogenic acid and rutin (Table 1) in wild-cultivated crosses of groundnut plants (*Arachis hypogaea* L. x *Arachis kempff-mercadoi* Krapov. and W.C. Greg.) were linked to their resistance to *Spodoptera litura* (Fab.) [86]. In carrot leaves, the flavone luteolin and the phenylpropanoid sinapic acid significantly differentiated thrip-resistant cultivars from susceptible ones (Table 1) [87]. In some cases of plant resistance to insects, the ratio of the specific metabolites was preponderant over their respective contents, as in carrot, where resistance to the fly *Psila rosae* F. is positively correlated with high luteolin-7-*O*-glucopyranoside/kaempferol-3-*O*-glucopyranoside and methyluteolin-7-*O*-glucopyranoside/kaempferol-3-*O*-glucopyranoside ratios [88].

Modes of action were investigated either in artificial bioassays or in planta. Chlorogenic acid was shown to significantly injure larval growth rate and larval survival capacity of *F. occidentalis* thrips when fed artificial diets including 5% chlorogenic acid (Table 1) [85]. Quercetin mostly contained in leaf extracts of castor beans (*Ricinus communis* L.) caused the death of adults and had remarkable ovicidal and oviposition-deterrent activity against *Callosobruchus chinensis* L., a common species of beetle found in the bean weevil subfamily and known to be a pest to many stored legumes (Table 1) [89]. Some compounds do not have direct insecticidal activity, but their physical and chemical properties can improve the solubility of other compounds and thus their penetration and efficacy. This sort of synergy was illustrated with non-PPP metabolites in an in vitro analysis that revealed an up to 19-fold increase in penetration of camphor in a binary mixture with 1,8-cineole through the larval integument of the cabbage looper (*Trichoplusia ni*) in comparison to camphor alone, the most toxic ratio being 60:40 1,8-cineole:camphor (LD_50_ = 186.9 μg/insect) [90,91]. Such a synergy could probably be searched within PPP metabolites. As mentioned before for fungal targets, one metabolite may have opposite actions against different targets, e.g., chlorogenic acid is on the one hand auxiliary to control thrips but on the other hand is promoting oviposition on carrot leaves by the black swallowtail (*Papilio polyxenes* Fabr.) [92]. An integrative approach is therefore needed to ensure that the protection strategy against one target does not increase severity of the disorders caused by another agent.

### 3.2. Vector Targets

Not only can insects cause direct damage to plants but they can also be vectors transmitting economically threatening diseases, such as Pierce’s disease, caused by the bacterium *Xyllella fastidiosa* [93], the grapevine yellow disease caused by phytoplasmas [94] or other major crop viruses. Therefore, limiting an epidemic by preventing the contact of the host with disease-carrying vectors is a major preoccupation in crop management. In this regard, Su and collaborators [95] addressed this dimension by linking metabolic changes in tomato leaves to vector behavior. They showed that whiteflies (*Bemisia tabaci*) actively recognized plants previously attacked by conspecifics due to decreased terpenoid and flavonoid contents. By treating tomato plants infested by *B. tabaci* with naringenin they increased their content of rutin, kaempferol-rhamnopyranoside, quercetin-trisaccharide, 3-*O*-methylmyricetin and anthocyanin up to the same level as those measured in noninfested plants and showed that the preference of *B. tabaci* for oviposition on previously infested plants was reversed (Table 1). As a consequence of reduced *B. tabaci* population, both the pest and the vectored virus—e.g., the tomato yellow leaf curl virus (TYLCV)—damage can be decreased. Moreover, whiteflies fed less on the phloem of flavonoid-rich tomato leaves, and the spread of TYLCV was reduced [96]. The authors pointed out that their findings rather suggest an impediment of the host–vector interaction than an antiviral activity of flavonoids, as disease expression was only delayed. Similarly, flavonoid accumulation was observed after infection of grapevine by the Flavescence dorée phytoplasma. This flavonoid accumulation was thought to repel the insect vectors afterwards [97].

The genes of the PPP involved in plant resistance to insects, considered direct herbivores or disease vectors, are poorly documented. Susceptibility of carrot roots to larval damage caused by the fly *Psila rosae* correlated with semiquantitatively estimated accumulation of *PAL1* and *PAL3* mRNAs in leaves [88]. The two genes responsible for their biosynthesis were expressed at a higher level in resistant lines than in susceptible ones. The PPP genes whose overexpression was correlated with the metabolic changes described above in the tomato–*B. tabaci* experiment [95] were the genes coding for *CHS, CHI*, flavonol synthase (*FLS*) and *DFR*.

In the field of drug discovery, phenylpropanoids and flavonoids are increasingly explored to develop eco-friendly insecticides targeting the vectors of major human diseases, such as mosquitoes [98,99,100,101,102,103]. At a time when resistance to conventional insecticides is alarming, recent works aimed at improving insecticide efficiency by overcoming the resistance mechanisms of insects. As a matter of fact, the activity of the CYP6AA3 and CYP6P7 cytochrome P450 monooxygenases of mosquito—known to detoxify insecticides such as pyrethroids—was inhibited by four flavones (apigenin, 5-hydroxy-7,8-dimethoxyflavone, 5-hydroxy-7,8,2′,3′-tetramethoxyflavone, and 5,4′-dihydroxy-7,8,2′,3′-tetramethoxyflavone) from *Andrographis paniculata* Nees (Table 1) [104]. Other studies focused more on the insect’s vital component and have investigated the neurotoxicity of plant phenylpropanoids to the target insects. On this basis, the mortality of *Aedes aegypti* was attributed to the inhibition of acetylcholinesterase activity by the phenylpropanoids asaricin, isoasarone and trans-asarone from *Piper sarmentosum* Roxb. leaf extracts (Table 1) [105]. This strategy may deserve to be explored for crop protection against insects.

## 4. Prospects

**Figure 2:**
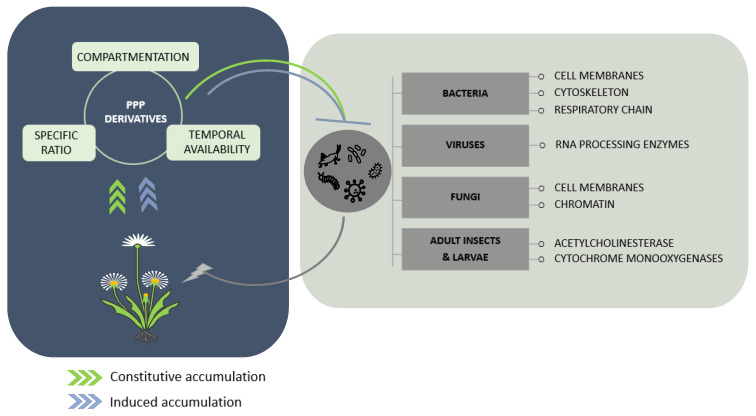
Potential intracellular targets of flavonoids and phenylpropanoids in microorganisms and insects aggressing plants. Biosynthetic accumulation can be constitutive and/or induced in response to external stimuli. The protective action of phenylpropanoids and flavonoids is described as the combinatory effect of their localization during the host’s interaction with aggressors, their sustained availability, and the promotion of specific compounds over others among the same subfamily or their putative synergy. Their action on microorganisms and insects probably arises from interference with important cellular machineries and structures, but this is not fully understood for all types of pests and pathogens.

The health benefits conferred by some plant-derived foodstuffs and beverages have greatly encouraged the exploration of the bioactive compounds involved and enhanced the investigation of their production in plant tissues [106,107,108]. This has paved the way for the understanding of the mechanisms of action of some phenylpropanoid derivatives on human bacterial and fungal agents, but also on viruses [76,82,83,109]. On this basis, knowledge from medical research could be a pioneer in understanding how plant-derived bioactive compounds negatively influence plant pathogen development (Figure 2).

An overview of the breakthroughs cited in this review clearly shows that some molecules exhibit a universal action regardless of the nature of the pathogen (summarized in Table 1). Chlorogenic acid, quercetin and other flavonoids are most frequently mentioned [55,56,76,89,95]. This universality has been exploited in the development of multidisease-resistant crops mediated by phenylpropanoids and flavonoids, e.g., myricetin (a flavonol) from tomato, to a vast array of herbivore insects [110]. Further described in [43], defense priming by rutin application on different host plants helped inducing SA-mediated defense responses against various bacterial pathogens. Similarly, quercetin induced the expression of defense-related genes in apple fruits [111]. Supporting these findings, the recent characterization of a gene in the PPP of maize corroborated the place held by these families of compounds: phenylpropanoids and flavonoids have become a promising and sustainable source of multiresistance [112]. However, despite this apparent potential in the expression of multiresistance, their action appeared to be more complex, as their accumulation in a given host can be perceived differently by various pathogens [41,92]. A preliminary systemic investigation based on metabolite contents should be undertaken prior to their selection for disease resistance. Efforts are still to be made in plant health research to understand the mechanisms of action of this family of compounds to better use them. Finally, it will be necessary to evaluate not only the content of metabolites of interest but also their location in the plant organs. Indeed, at the tissue level, phenylpropanoids can be found in grain pericarps and in various leaf tissues, such as glandular trichomes, cuticle, epidermis and mesophyll [25,36,113,114]. Immunolocalization of *PAL* and *CHS* in *Primula kewensis* W. Wats. suggested that flavonoid biosynthesis occurred in the head of glandular cells [115]. The concentration in aglycones and their glycosides may vary, considering different levels of the leaf tissue, suggesting the specificity of their function [116]. At the cellular level, phenylpropanoid derivatives are stored in vacuoles, but can also be detected in the cell wall [117]. This accumulation inside or at the peripheral sites of the cell/tissue/organs may signify the formation of physical or chemical barriers preventing pathogen or pest invasion (direct effect) or an involvement in plant signaling to mediate defense responses or plant-to-plant communication. Therefore, depending on the mechanisms of action, the metabolite content required to be efficient for plant protection may be different.

**Table 1:** Overview of main active PPP compounds and their putative mechanisms of action. Molecules are sorted in two families, phenylpropanoids and flavonoids, and are alphabetically presented within the families. The table does not include all the compounds cited in the text, but only those that have been documented either through genetic studies on segregating progenies, functional validation of candidate genes explaining their biosynthesis, direct toxicity tests on the targets, or histological observations of the effects of the compound on the target. Compounds for which the literature only relates a correlation between resistance level and metabolite content are not described. Concentration range is given for the compounds with biological activity reported in the corresponding references. MIC, minimum inhibitory concentration; EC_50_, half-maximal effective concentration; IC_50_, half-maximal inhibitory concentration; MC_50_, half larval mortality concentration.

Compound	Host	Targets	Concentration	Mechanisms	Ref.
Phenylpropanoids					
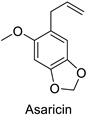	*Piper sarmentosum*	*Aedes aegypti*	IC_50_ = 0.73 µg·mL^−1^	Inhibition of acetylcholinesterase activity	[105]
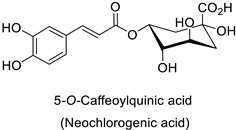	Tobacco (*Nicotiana tabacum*)	Tobacco mosaic virus (TMV)	-	Accumulation at the infection site	[11]
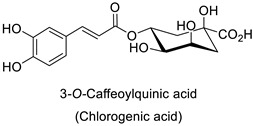	Human	*Escherichia coli*	IC_50_ = 69.55 ± 3.6 µM	Inhibition of FtsZ polymerase (a cell division protein)	[55]
		*Shigella* *dysenteriae*	MIC = 20 µg·mL^−1^	Bacterial outer membrane disintegration	[52]
		*Candida* *albicans,* *Trichosporon beigelii* and *Malassezia* *furfur*	MIC = 40–80 µg·mL^−1^	Deterioration of membrane electrical potential	[76]
	Groundnut *(Arachis hypogaea)*	*Spodoptera litura* (Fab.)	* MC_50_ = 0.67 µg·mL^−1^	Larvicidal activity or limitation of larval development and adult malformation	[86]
	Chrysanthemum (*Dendranthema* *grandiflora*)	*Frankliniella* *occidentalis*	5% in culture medium	Reduction of larval growth rate and adults survival rate	[85]
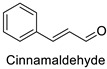	Tomato *(Solanum* *lycopersicum)*	*Alternaria* *alternata*	MIC = 800 µg·mL^−1^ (Essential oil containing 30% of cinnamaldehyde and 44% of eugenol)	Cell wall invaginations and distortions	[75]
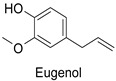	Tomato *(Solanum* *lycopersicum)*	*Alteranaria* *alternata*	MIC = 800 µg·mL^−1^ (Essential oil containing 30% of cinnamaldehyde and 44% of eugenol)	Cell wall invaginations and distortions	[75]
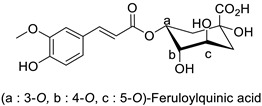	Chrysanthemum (*Dendranthema* *grandiflora*)	*Frankliniella* *occidentalis*	5% in culture medium	Reduction of larval growth rate and adults survival rate	[85]
	Carrot *(Daucus carota)*	*Alternaria dauci*	-	-	[57]
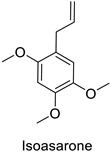	*Piper sarmentosum*	*Aedes aegypti*	IC_50_ = 0.92 µg·mL^−1^	Inhibition of acetylcholinesterase activity	[105]
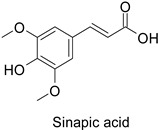	Carrot (*Daucus carota*)	*Psila rosae*	0.35–2.11 mg·g^−1^	Insecticidal activity	[87]
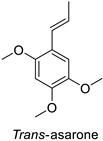	*Piper sarmentosum*	*Aedes aegypti*	IC_50_ = 15.75 µg·mL^−1^	Inhibition of acetylcholinesterase activity	[105]
Flavonoids					
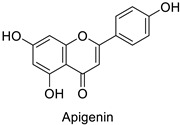	Human	*Candida albicans*	5µg·mL^−1^	Cell shrinkage due to membrane disruption	[77]
		*Anopheles* *minimus*	IC_50_ = 2.38 µM	Inhibition of cytochrome P450 monooxygenases	[104]
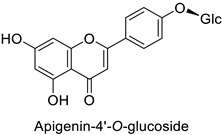	Carrot (*Daucus carota*)	*Alternaria dauci*	-	-	[57]
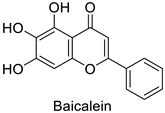	Human	Murine leukemia viruses (MLVs)	90% of inhibition at 1 µg·mL^−1^	Inhibition of reverse transcriptase activity	[82]
		Human immunodeficiency viruses (HIVs)	90% of inhibition at 2 µg·mL^−1^		
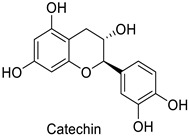	Cotton (*Gossypium* *hirsutum* and *Gossypium barbadense)*	*Verticillium dahliae*	-	Confinement of the pathogen at the infection site	[61]
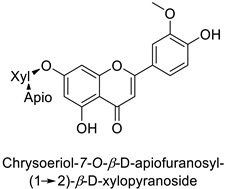	Human	*Staphylococcus aureus*	MIC = 4–8 µg·mL^−1^	Cytoplasmic membrane disruption	[48]
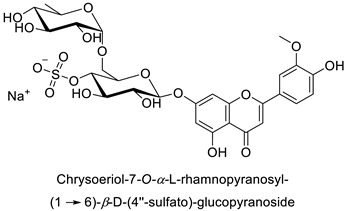					
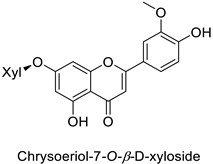					
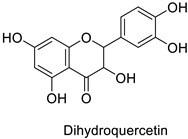	-	*Fusarium* *graminearum*	IC_50_ = 124.27 mg·L^−1^	Mycelial growth inhibition	[74]
	-	*Septoria zeicola*	IC_50_ = 160.32 mg·L^−1^		
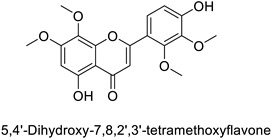	Human	*Anopheles* *minimus*	IC_50_ = 5.91–16.6 µM	Inhibition of cytochrome P450 monooxygenases	[104]
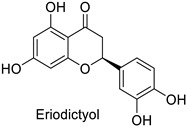	-	*Fusarium* *graminearum*	IC_50_ = 162.71 mg·L^−1^	Mycelial growth inhibition	[74]
	-	*Septoria zeicola*	IC_50_ = 247.32 mg·L^−1^		
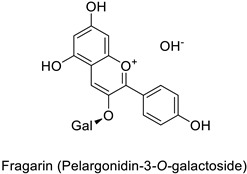	Strawberry (*Fragaria ananassa*)	*Clavibacter* *michiganensis*	EC_50_ = 0.07 µM	Cell membrane permeabilization	[44,45]
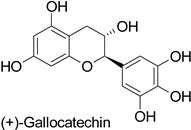	Cotton (*Gossypium hirsutum* and *Gossypium barbadense)*	*Verticillium dahliae*	-	Confinement of the pathogen at the infection site	[61]
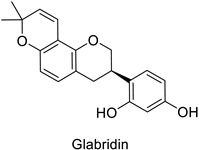	Human	*Candida glabrata*	MIC = 4–16 µg·mL^−1^	DNA fragmentation and chromatin condensation	[78]
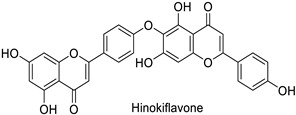	Human	Human immunodeficiency virus	IC_50_ = 62 µM	Inhibition of reverse transcriptase activity	[83]
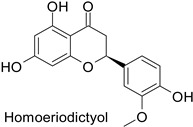	-	*Fusarium* *graminearum*	IC_50_ = 274.78 mg·L^−1^	Mycelial growth inhibition	[74]
	-	*Septoria zeicola*	IC_50_ = 240.31 mg·L^−1^		
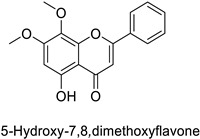	Human	*Anopheles* *minimus*	IC_50_ = 7.24–8.90 µM	Inhibition of cytochrome P450 monooxygenases	[104]
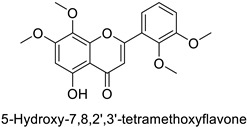			IC_50_ = 6.45–8.35 µM		
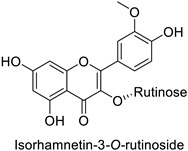	Human	*Staphylococcus aureus*	MIC = 4–8 µg·mL^−1^	Cytoplasmic membrane disruption	[48]
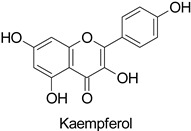	Tobacco (*Nicotiana tabacum*)	Tobacco mosaic virus (TMV)	-	Accumulation at SAR site	[11]
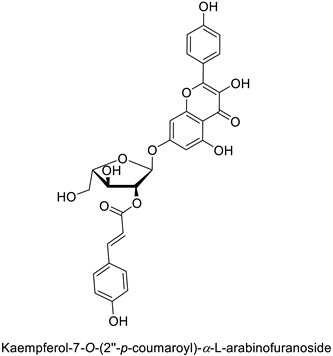	Pine tree (*Picea neoveitchii*)	*Rhizoctonia solani*	49.5% of growth inhibition at 1 mg·mL^−1^	Mycelial growth inhibition	[58]
		*Fusarium* *oxysporum*	108.1% of growth inhibition at 1 mg·mL^−1^		
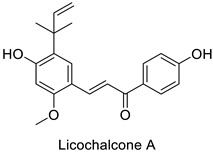	Human	*Micrococcus* *luteus*	MIC = 1.56 µg mL^−1^	Inhibition of NADH-cytochrome C reductase	[51]
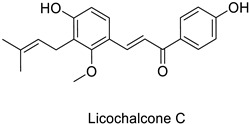			MIC = 6.25 µg mL^−1^		
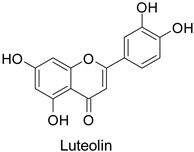	Carrot (*Daucus carota*)	*Psila rosae*	0.35–2.11 mg·g^−1^	Insecticidal activity	[87]
	-	*Fusarium* *graminearum*	IC_50_ = 56.38 mg·L^−1^	Mycelial growth inhibition	[74]
	-	*Septoria zeicola*	IC_50_ = 81.48 mg·L^−1^		
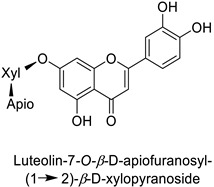	Human	*Staphylococcus* *aureus*	MIC = 4–8 µg·mL^−1^	Cytoplasmic membrane disruption	[48]
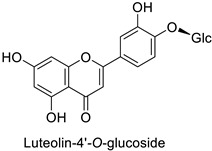	Carrot (*Daucus carota*)	*Alternaria dauci*	-	-	[57]
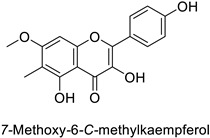	Pine tree (*Picea neoveitchii*)	*Rhizoctonia solani*	95.3% of growth inhibition at 1 mg·mL^−1^	Mycelial growth inhibition	[58]
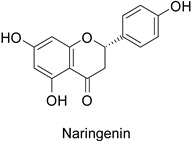	Human	*Staphylococcus aureus*	MIC = 1.84 mM	Membrane fatty acid rearrangement leading to membrane integrity and fluidity alteration.	[49]
		*Escherichia coli*	MIC = 3.64 mM		
	Tomato *(Solanum lycopersicum)*	*Bemisia tabaci*	10 µM	Oviposition deterrent	[95]
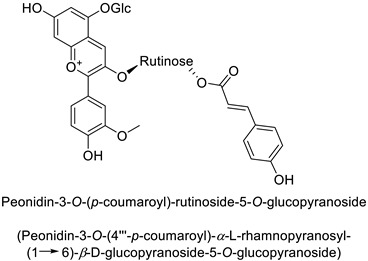	Potato *(Solanum tuberosum)*	*Erwinia* *carotovora*	-	-	[42]
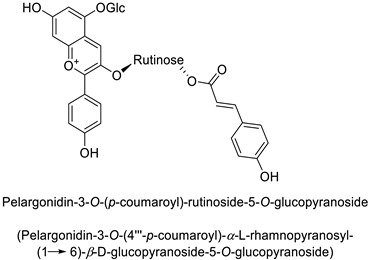					
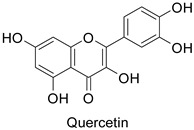	Tobacco (*Nicotiana tabacum*)	Tobacco mosaic virus (TMV)	-	Accumulation at the infection site	[11]
	*Datura stramonium* and *Chenopodium* *amaranticolor*		250 µM	Putative host defense mediation through salicylic acid biosynthesis	[81]
	Groundnut *(Arachis hypogaea*)	*Spodoptera litura* (Fab.)	* MC_50_ = 0.73 µg·mL^−1^	Larvicidal activity or limitation of larval development and adult malformation	[86]
	Stored legumes	*Callosobruchus chinensis* L.	3 mg·mL^−1^ (leaf extract mainly containing quercetin)	Insecticidal activity	[89]
			6 mg·mL^−1^ (leaf extract mainly containing quercetin)	Oviposition deterrent Ovicidal activity	
	*Datura stramonium* and *Chenopodium* *amaranticolor*	Tobacco mosaic virus (TMV)	250 µM	Putative host defense mediation through kaempferol and salicylic acid biosynthesis	[81]
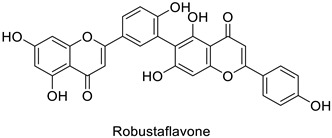	Human	Human immunodeficiency virus	IC_50_ = 65 µM	Inhibition of reverse transcriptase activity	[83]
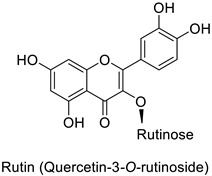	Potato (*Solanum tuberosum*)	*Pectobacterium atrosepticum*	88 µg·mL^−1^	-	[41]
	Groundnut *(Arachis hypogaea*)	*Spodoptera litura* (Fab.)	* MC_50_ = 0.60 µg·mL^−1^	Larvicidal activity or limitation of larval development and adult malformation	[86]
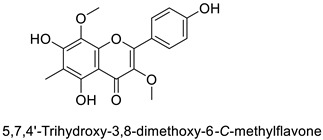	Pine tree (*Picea neoveitchii*)	*Rhizoctonia solani*	49.5% of growth inhibition at 1 mg·mL^−1^	Mycelial growth inhibition	[58]
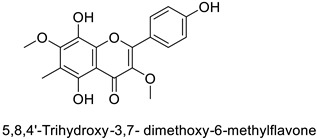			53.3% of growth inhibition at 1 mg·mL^−1^		
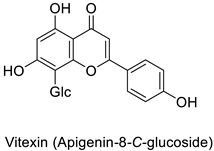	*Datura stramonium* and *Chenopodium* *amaranticolor*	Tobacco mosaic virus (TMV)	250 µM	Putative host defense mediation through kaempferol and salicylic acid biosynthesis	[81]

* According to linear multiple regression equation.
